# Genomic analysis of mouse VL30 retrotransposons

**DOI:** 10.1186/s13100-016-0066-8

**Published:** 2016-05-06

**Authors:** Georgios Markopoulos, Dimitrios Noutsopoulos, Stefania Mantziou, Demetrios Gerogiannis, Soteroula Thrasyvoulou, Georgios Vartholomatos, Evangelos Kolettas, Theodore Tzavaras

**Affiliations:** Laboratory of General Biology, Faculty of Medicine, School of Health Sciences, University of Ioannina, Ioannina, 45110 Greece; Biomedical Research Division, Institute of Molecular Biology and Biotechnology, Foundation of Research and Technology (IMBB-FORTH), University Campus, Ioannina, 45110 Greece; Laboratory of Molecular Biology and Genetics, Department of Biological Applications and Technology, School of Health Sciences, University of Ioannina, Ioannina, 45110 Greece; Department of Computer Science, School of Sciences, University of Ioannina, Ioannina, 45110 Greece; Hematology Laboratory, Unit of Molecular Biology, University Hospital of Ioannina, Ioannina, 45110 Greece

**Keywords:** VL30s, Retrotransposon, PSF, Mouse genome, Exaptation

## Abstract

**Background:**

Retrotransposons are mobile elements that have a high impact on shaping the mammalian genomes. Since the availability of whole genomes, genomic analyses have provided novel insights into retrotransposon biology. However, many retrotransposon families and their possible genomic impact have not yet been analysed.

**Results:**

Here, we analysed the structural features, the genomic distribution and the evolutionary history of mouse VL30 LTR-retrotransposons. In total, we identified 372 VL30 sequences categorized as 86 full-length and 49 truncated copies as well as 237 solo LTRs, with non-random chromosomal distribution. Full-length VL30s were highly conserved elements with intact retroviral replication signals, but with no protein-coding capacity. Analysis of LTRs revealed a high number of common transcription factor binding sites, possibly explaining the known inducible and tissue-specific expression of individual elements. The overwhelming majority of full-length and truncated elements (82/86 and 40/49, respectively) contained one or two specific motifs required for binding of the VL30 RNA to the poly-pyrimidine tract-binding protein-associated splicing factor (PSF). Phylogenetic analysis revealed three VL30 groups with the oldest emerging ~17.5 Myrs ago, while the other two were characterized mostly by new genomic integrations. Most VL30 sequences were found integrated either near, adjacent or inside transcription start sites, or into introns or at the 3′ end of genes. In addition, a significant number of VL30s were found near Krueppel-associated box (KRAB) genes functioning as potent transcriptional repressors.

**Conclusion:**

Collectively, our study provides data on VL30s related to their: (a) number and structural features involved in their transcription that play a role in steroidogenesis and oncogenesis; (b) evolutionary history and potential for retrotransposition; and (c) unique genomic distribution and impact on gene expression.

**Electronic supplementary material:**

The online version of this article (doi:10.1186/s13100-016-0066-8) contains supplementary material, which is available to authorized users.

## Background

Mobile DNA sequences occupy almost half of many mammalian genomes [1, 2], and in the past they have been considered as solely selfish or junk DNA sequences. Recent studies, however, have provided evidence that mobile DNA can be harnessed for host functions, and it is now accepted that mobile DNA-host interactions play a major role in organism physiology, pathology and evolution [3]. Mobile DNA elements are divided into two major groups, DNA transposons and retrotransposons. While DNA transposons mobilize through a conservative mechanism, comprising their excision and subsequent integration into a new site, retrotransposon numbers increase in the genome through an RNA intermediate via a mechanism known as retrotransposition [3] and, based on their genomic structure, they are further subdivided into Long Terminal Repeat (LTR)- and non-LTR retrotransposons.

Viral-like 30 elements (VL30s) are a family of 5–6 Kb retrovirus-like DNA sequences present in mouse and rat genomes, and are classified as LTR retrotransposons [4]. VL30 LTRs contain a large repertoire of transcription factor binding sites that provide a remarkable plasticity for VL30 RNA expression [4, 5]. VL30 transcription is tissue-specific [6], up-regulated by histone phosphorylation, acetylation and DNA demethylation [7], and can be induced by several different factors including C2 ceramide, a dominant negative p53 oncoprotein [8] or in cerebral ischemia [9]. VL30s are considered as immediate early response genes as their RNA expression is rapidly induced following transient inhibition of protein synthesis or mitogenic stimulation [10, 11]. Another important feature of VL30s is that their RNA is packaged into C-type retroviral particles of murine leukemia viruses and can be transmitted to heterologous cells [12, 13], making them an important potential tool for gene transfer. VL30 transcripts play functional roles as inducible VL30 transcription regulates expression of neighboring genes, creating a transcription network [14]. Induced VL30 RNA seems to be a critical factor involved in oncogenesis and steroidogenesis [15, 16], as it contains specific motifs for binding to spliceosome factor (PSF), leading to RNA induction of PSF-repressed genes.

Sequence analysis of two VL30 members, NVL-3 [17] and BVL-1 [18], has revealed no coding open reading frames due to multiple stop codons for *gag* and *pol*, and no evidence for *env* retroviral genes, classifying them as non-autonomous LTR retrotransposons. Specifically, the NVL-3 member is retrotransposition-competent [19, 20] and its retrotransposition is induced by the large T antigen of Simian virus 40 [19] or oxidative stress agents such as hydrogen peroxide [21], vanadium [22] and arsenic [23]. Finally, induced VL30 retrotransposition activates a caspase-independent and p53-dependent cell death pathway associated with mitochondrial and lysosomal damage [24].

The availability of bioinformatics tools allows the analysis of genomic DNA sequences related to their genomic distribution, evolution and prediction of functional properties. Such analyses have revealed that more than 2/3 of mammal genomes may be comprised of mobile DNA [25], a much higher percentage than the ~50 % initially calculated [1]. They have also provided novel insights on the features and biology of LTR retrotransposons such as human HERV-Ks [26] and many mouse LTR retrotransposons [27, 28].

Data on mouse VL30s comes from studies based on conventional methods, such as hybridization analysis and sequencing [4, 5], and gross estimations [29], prior to publication of the mouse genome, but a detailed genomic analysis of mouse VL30s is not yet available. In this study, we systematically annotated VL30 sequences and analyzed their number, structure, phylogeny and genomic distribution providing new insights for their potential role in the mouse genome.

## Results

### Copy number, structural features and variation of VL30 sequences

As the only available genomic data for VL30s come from sequencing of a few individual elements [4], prior to publication of the mouse genome, we attempted to measure their total number, analyse their structural features and predict functional traits of particular VL30 sequences.

To search for VL30 sequences in the UCSC mouse Database [30, 31], we assumed that: (i) an intact VL30 element, independent of its length, should contain near identical 5′ and 3′ LTRs, an intact retroviral Primer Binding Site (PBS) required for binding of a primer tRNA to initiate reverse transcription, and a polypurine Tract (PPT) responsible for initiating (+) strand synthesis during reverse transcription; (ii) a truncated VL30 should contain an internal sequence but not necessarily two intact LTRs; and (iii) solo LTRs, of varying sequence length, should only contain LTR sequences as defined by the Repeat masker tool [32]. Using a combination of Repeat Masker analysis (http://www.repeatmasker.org) for VL30 internal sequences [32], BLAT querying [33] for known VL30 consensus sequences from Repbase [34] and *in-silico* PCR for specific VL30 primers, corresponding to a conserved internal region [35], our genomic search initially revealed 372 total VL30 sequences. All sequence data were extracted along with 2000 bp of 5′ and 3′ flanking genomic regions.

Using the LTR-finder tool [36] in the above extracted VL30 sequence data we distinguished full-length from truncated and solo LTR sequences and found that the mouse genome contains 86 full-length elements in a sequence length ranging from 4075 to 6321 bp. In addition, manual annotation of the remaining non-intact VL30 sequence data revealed 49 truncated elements and 237 relative solo LTRs. The particular genomic coordinates of full-length, truncated and solo LTR VL30s as well as their sequence features are provided in Additional file 1.

**Fig. 1:**
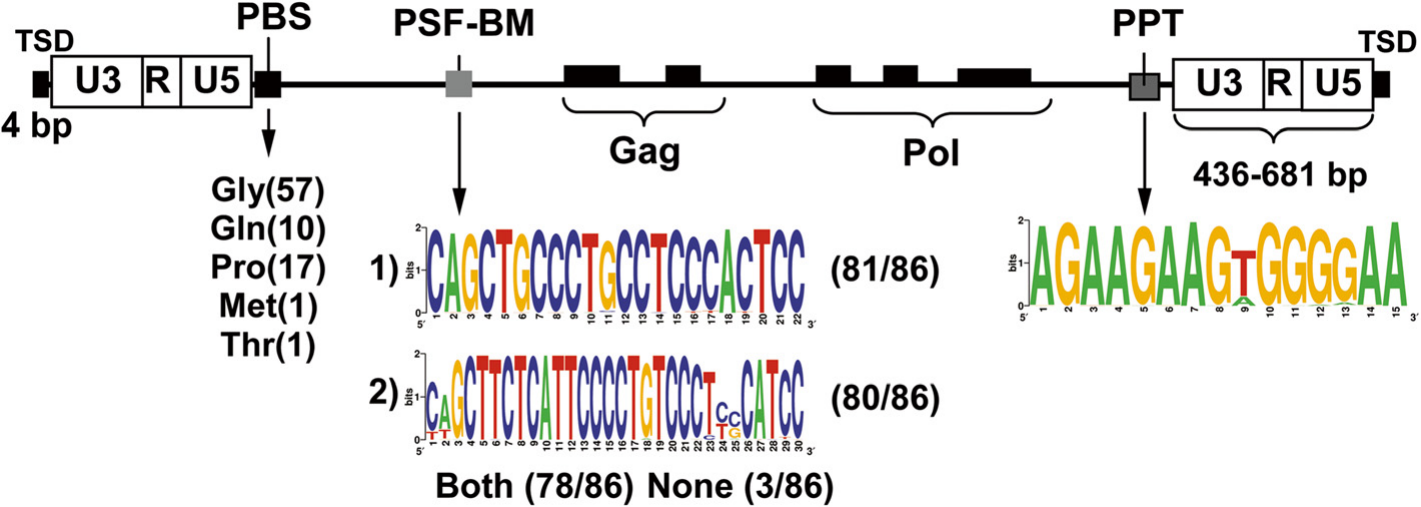
Structural properties of full-length VL30 elements. A representative VL30 element is depicted, using sequence data from UCSC genome browser, LTR-finder and additional BLAST searches. Depicted from 5′ to 3′: LTRs: Long Terminal Repeats, PBS: Primer Binding Site; PSF-BM: PSF Binding Motif; Gag, Pol: regions related to retroviral genes (but contain multiple stop-codons); PPT: Poly-Purine Tract; TSD: Target Site Duplication. Gly, Gln, Pro, Met and Thr indicate tRNA species complementary to PBS signals of VL30 elements, while in parentheses is shown the number of elements that use each tRNA. Below PSF-BM are presented the sequences of the two specific motifs of PSF binding, while below PPT region the 15-bp consensus sequence

Next, we examined the full-length VL30s for variations in their LTR sequences and retroviral replication signals. It was found that LTRs were quite heterogeneous in sequence length ranging from 436- to 681 bp (Fig. 1). In keeping with this, an LTR-related structural feature of retroviruses are Target Site Duplications (TSDs), a few bp long, generated following integration of their LTRs into the genome. Hence, we attempted to analyze the TSDs of full-length VL30 sequences found, using the LTR-finder tool [36]. The examination of mouse genomic sequences, immediately adjacent to VL30 LTRs, showed TSDs in 76 out of 86 full-length elements with a random 4-bp long motif (Additional file 1). The 86 full-length elements were distinguished by heterogeneous 15–20 bp long PBS signals, allowing binding of various tRNAs. Specifically, 57 elements contained PBS signals corresponding to Gly tRNA species, while a distinctively smaller number of 17 and 10 elements contained PBS signals corresponding to Pro and Gln tRNA species, respectively. Only two elements contained Met or Thr tRNA species. Further analysis revealed that all full-length elements had a PPT region, which was highly conserved in 68 elements containing the 15-bp consensus sequence AGAAGAAGTGGGGAA, while the rest 18 elements had one or two A/T substitutions (Fig. 1 and Additional file 1).

Early studies have shown that mouse and rat VL30 RNAs can be efficiently packaged into MoMLV virions [37, 38] through a retroviral Ψ packaging-sequence signal. Given that a mouse VL30 Ψ consensus sequence is not known, we attempted to identify such a sequence comparing the full-length sequences with the known 68-bp purine-rich sequence GGCAAGCCGGCCGGCG [38], downstream of the rat 5′ LTR VL30, which is critical for RNA dimerization and packaging. Examining the entire internal sequence of all 86 full-length sequences by the Blast algorithm, between 5′ and 3′ LTRs as well as downstream of U5 and upstream of U3 regions, neither a conserved nor a rat-related Ψ signal motif was found.

Finally, based on the property of VL30 RNA to bind PSF [39], we analyzed, using Blast alignment, all full-length VL30 sequences for the two known specific motifs of PSF binding: CAGCTGCCCTGCCTCCCACTCC and CAGCTTCTCATTCCCCTGTCCCTCCCATCC [15]. We found that in contrast to three PSF-negative elements, five contained only one while all the rest of the 78 sequences contained both motifs (Fig. 1 and Additional file 1). Next, we asked whether truncated elements also had PSF binding motifs. A similar analysis showed that 9 out of 49 truncated elements had no PSF signal motifs. In contrast, 6 elements contained one while 34 contained both PSF-binding motifs (Additional file 1).

### VL30s transcriptional and protein coding potential

While both LTR sequences participate in the integration of a retrovirus into the host genome, the 5′ LTR serves as a promoter regulating the entire retroviral RNA expression accomplished through cellular transcription factors which interact with specific DNA motifs located in this region.

We attempted to identify transcription factor binding sites (TFBS) over the entire 5′ LTR sequences of full-length VL30s using the Matinspector tool [40] in the Genomatix database (see Methods). Specifically, we analyzed the group of 5′ LTRs of all 86 full-length VL30s found along with a group of 5′ LTRs of 7 known VL30 elements (such as NVL1/2, NVL3, BVL1, VL3, VM1, VL11 and B10) for common or unique TFBS. Our analysis revealed 10,371 TFBS of 53 distinct transcription factors (TFs) to exist in 70 % of both element groups and a total number of 15,039 (13,912 in 86 full-length elements, denoted in parenthesis thereof) TFBS of 197 TFs in at least one element (Additional file 2). The number of TF binding sites largely varied depending on the particular VL30 LTR and chromosome. For example, the binding sites of HOMF (homeobox-domain factor) and VTBP (Vertebrate TATA Binding Protein) were present in all 93 (86) (Additional file 2) analyzed LTRs and in all chromosomes with a high number of 550 and 370 sites (516 and 346) ranging between 1–12 and 1–7 per chromosome, respectively. To a lesser extent, the glucocorticoid responsive/related elements (GREF) and nuclear factor kappa-light chain enhancer of activated B cells (NF-κB) TF binding sites were present in 87 and 85 LTRs, with 265 and 131 binding sites, respectively, ranging 1–7 and 1–4 sites per chromosome. Finally, some TF binding sites were extremely infrequent with 1–2 sites restricted either to only one or two LTRs in a particular chromosome, such as those of HASF and MEF3 with two binding sites on 2 different chromosomes, or CABL and HNFP with 1 binding site in different chromosome, respectively (Additional file 2).

In addition, we analysed the truncated and solo LTR sequences since they can also act as promoters or enhancers, driving the expression of adjacent genomic loci. We scored 8558 and 44,428 total TFBS in truncated and solo LTR sequences, respectively. In analogy to full-length elements, HOMF and VTBP TFBSs we present in almost all truncated sequences with a high number of 238 (in 47 out of 49 sequences) and 122 (in 48 out of 49 sequences) sites, respectively. As regards solo LTRs the respective TFBSs for HOMF and VTBP were 1266 and 643 in 234 out of 237 sequences, respectively (Additional file 2).

Next, we extended our analysis searching for VL30-associated ESTs in the mouse section of dbEST. We retrieved 592 total ESTs starting or terminating within 99 distinct VL30 sequences. In particular, 414 different ESTs were identified in two-thirds of the full-length VL30 sequences (57 out of 86). Moreover, we found 85 and 93 ESTs in 14 truncated and 28 solo LTR sequences, respectively (Additional file 3).

In a final step, we searched whether VL30s have protein coding potential, using the ORF-finder [41] in the sequences of full-length elements. The analysis of their internal sequences between 5′ and 3′ LTRs, revealed multiple stop codons and no intact full-open reading frames corresponding to typical *gag*, *pol* and *env* retroviral genes (data not shown).

### The genomic distribution of VL30s

Taking into account the total number of 372 VL30 sequences found, we asked whether there are random or “hot spot” integrations of VL30s in the mouse genome, as their genomic distribution is unknown.

**Fig. 2:**
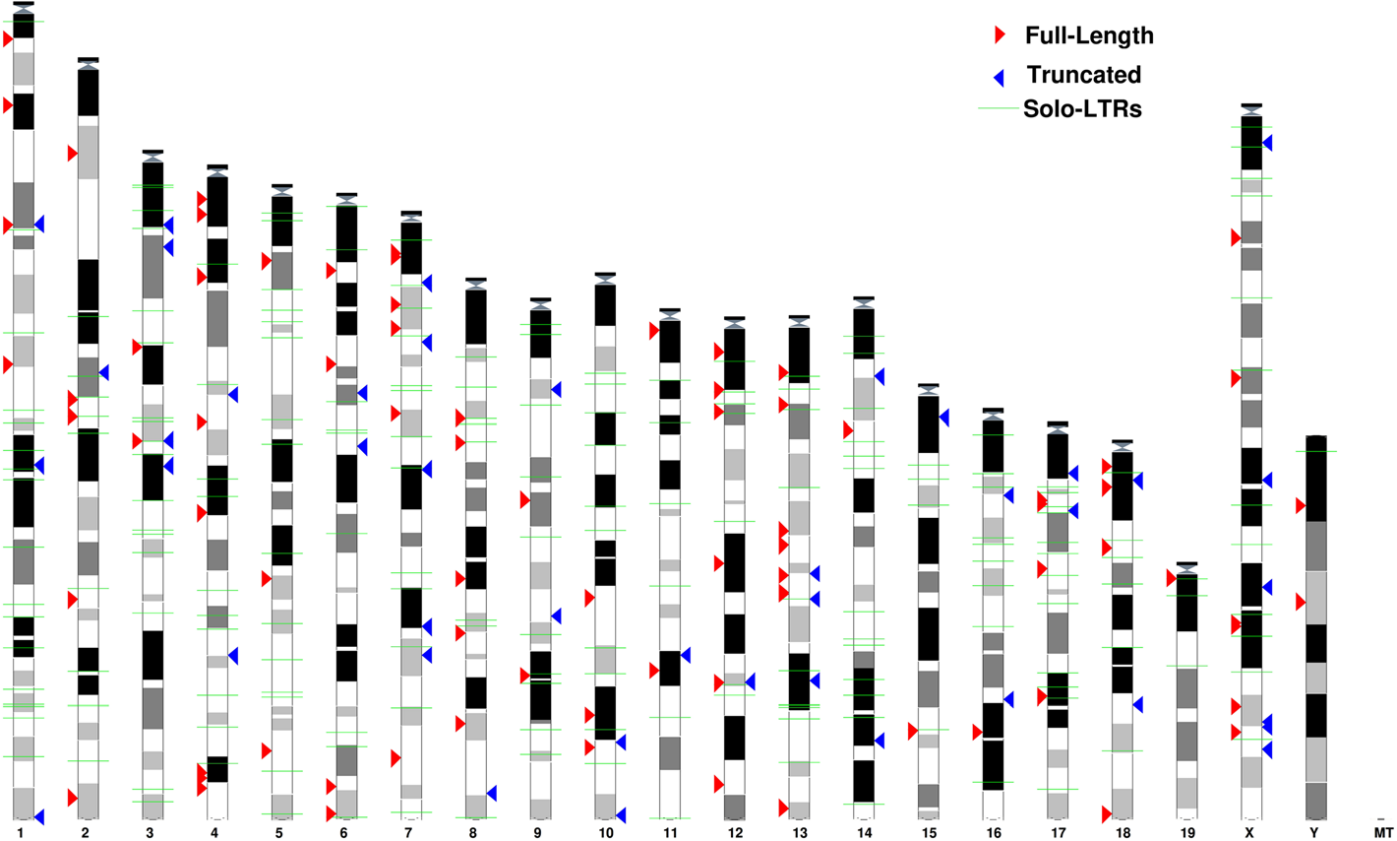
Chromosomal distribution of VL30 elements. Full-length VL30s, truncated VL30s and solo LTRs have been annotated to their respective genomic positions and projected to mouse chromosome ideograms, using the Ensembl Genome Browser. Red arrows denote full-length VL30s, blue arrows truncated elements and green horizontal lines solo LTRs

**Fig. 3:**
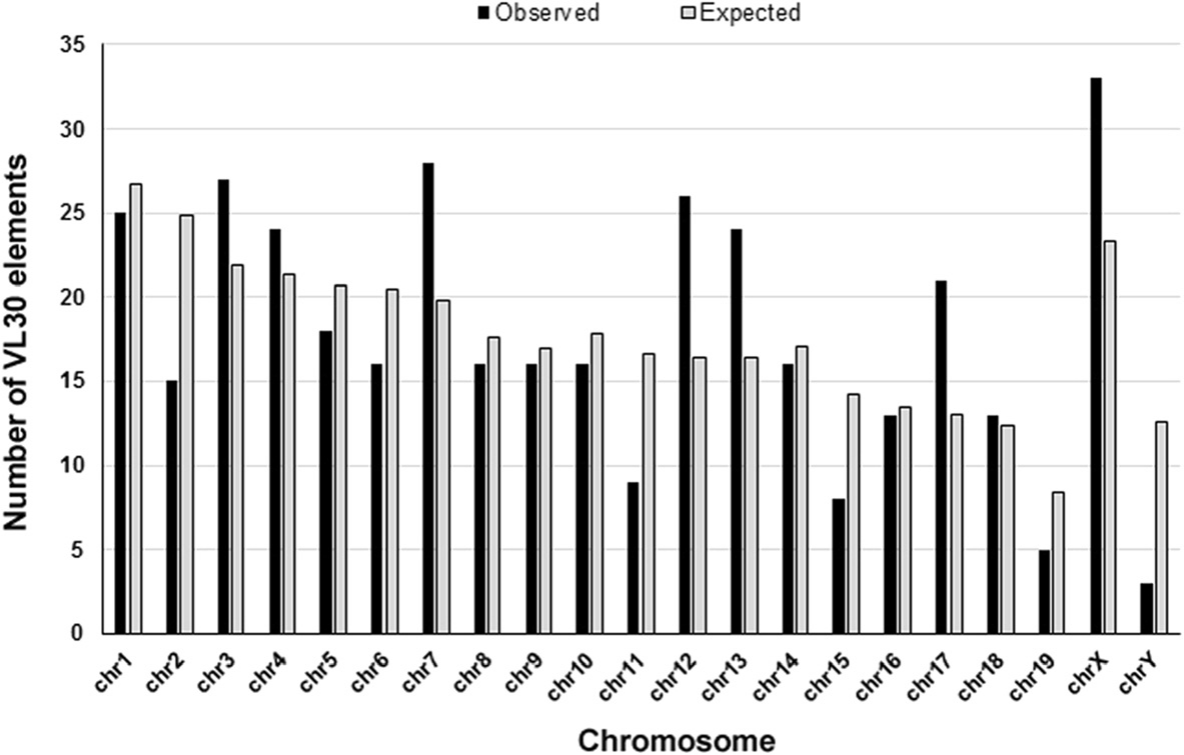
Mouse genomic distribution of VL30 elements. Histogram bars indicate the observed (black) and expected (light grey) number of VL30 elements from each mouse chromosome. The expected number of VL30 elements on each chromosome was calculated following multiplication of the chromosomal length with average density of VL30s in the mouse genome

**Fig. 4:**
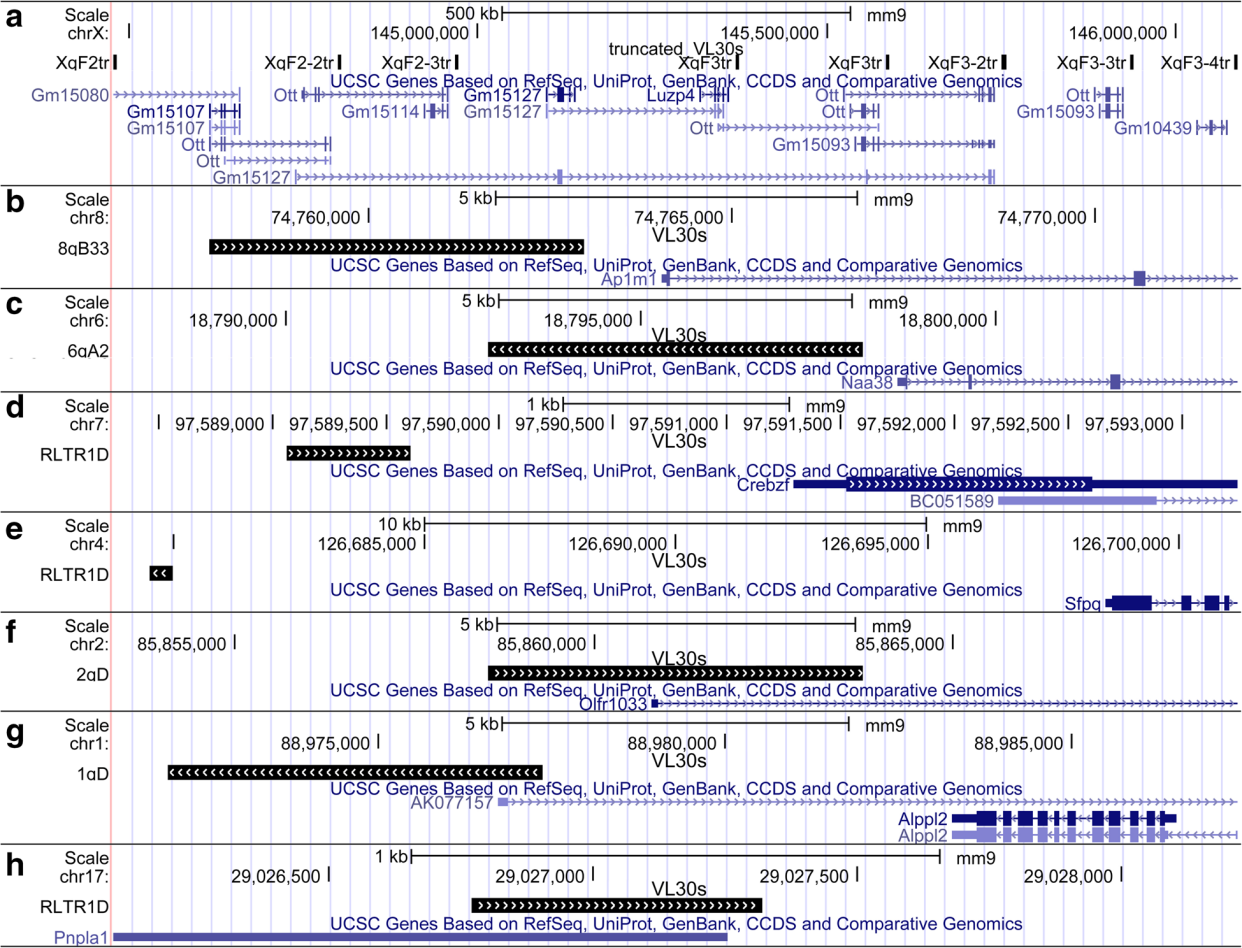
Examples of VL30 integration events with possible significance for the mouse genome. Genomic regions (panels **a**-**h**) containing integration events with a possible significance in the mouse genome were found after GREAT analysis and manually confirmed in UCSC Genome Browser. Genome graphs were extracted from the UCSC Genome Browser. The chromosome number, scale, VL30 element name and associated genes name in each region are shown

Using the Ensembl Genome Browser tool [42], we created a map representing the distribution of full-length, truncated as well as solo LTR VL30 sequences in the mouse chromosomes (Fig. 2). By this analysis we primarily found that all full-length, truncated and solo LTR sequences were represented in all 21 autosomal and sex chromosomes. Moreover, assuming a random insertion model based on chromosome size, we analyzed their distribution at the chromosomal level comparing the observed with the relative expected number of VL30 integrations. Statistical analysis was revealed a significant difference between the two distributions (chi-square = 43.66, df = 20, *p* <0.002). We found that VL30s density was much higher than expected on chromosomes 3, 7, 12, 13, 17 and X. In contrast, chromosomes 2, 11, 15, 19 and Y had fewer elements than expected (Fig. 3). In addition, we observed the presence of 8 almost identical truncated VL30s integrated in a particular genomic domain of 1.5 Mb at XqF2 and XqF3 regions of chromosome X (Fig. 4a). In a further step, we examined the GC content of VL30 integration sites. By analyzing the GC content of 400 bp genomic DNA flanking full-length VL30 sequences, we found that VL30s were integrated in genomic regions characterized by a ~42 % GC content (Additional file 4).

**Fig. 5:**
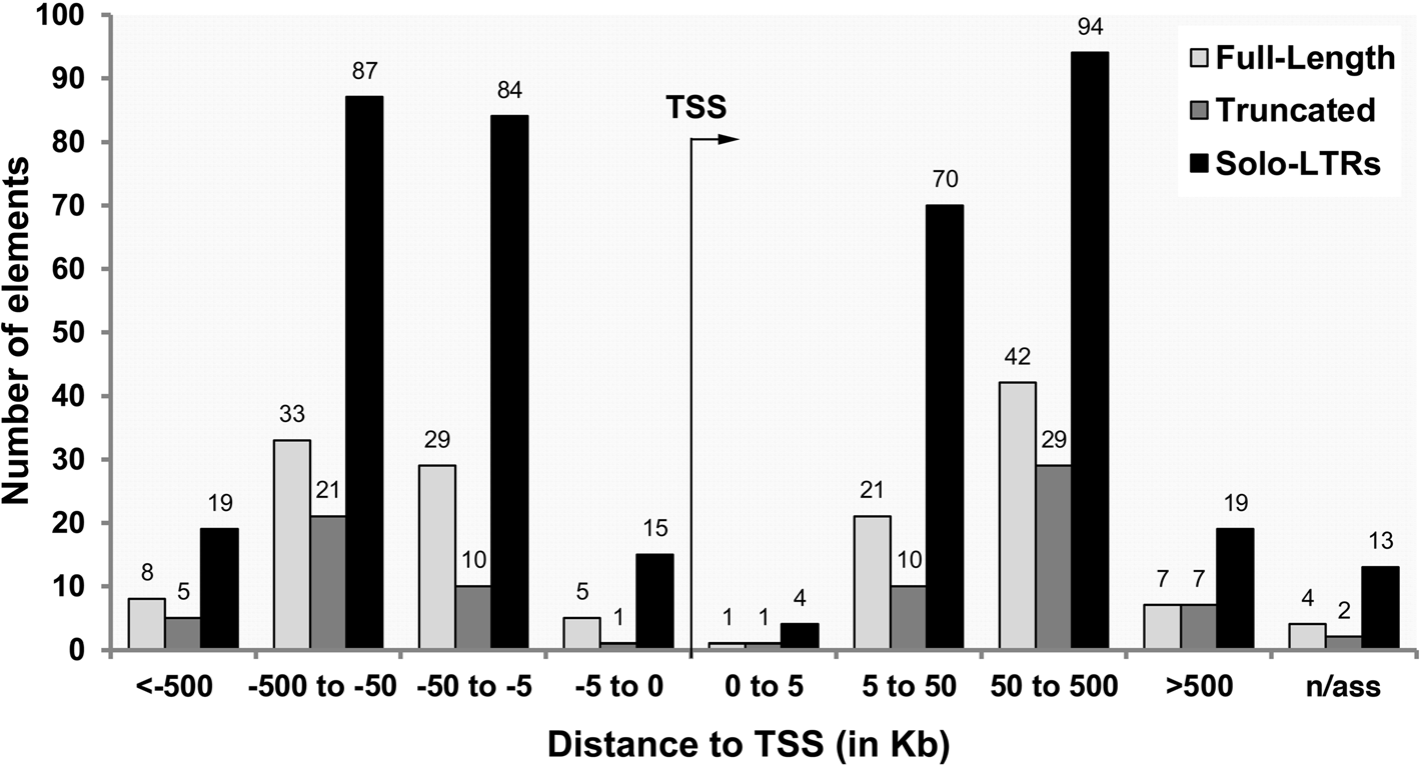
Distribution of VL30s relative to transcription start sites of mouse genes. Analysis with GREAT tool under default settings was performed for full-length, truncated VL30s and solo LTRs. The chart shows the relative distance (0–5, 5–50, 50–500, 500–1000 Kb) of full-length (light grey), truncated (dark grey) or solo LTRs (black) to Transcription Start Site (TSS). n/ass denotes VL30s not associated to any genes

Next, uploading the VL30 genomic coordinates (Additional file 1) into the GREAT annotation tool [43], we analyzed the distribution of VL30 sequences in relation to mouse gene regions. Out of 372 total VL30 sequences, 317 sequences were integrated up to 500 kilobases upstream of transcription start sites (TSS). Specifically, 33 full-length, 21 truncated and 87 solo LTR sequences were found at a distance between −500 and −50 kilobases upstream of TSS; 29 full-length, 10 truncated and 84 solo LTR sequences at a distance between −50 and −5, while 5 full-length, 1 truncated and 15 solo LTR sequences were the nearest integrated sequences upstream of TSSs. Moreover, 107 total sequences: full-length, truncated and solo LTRs were integrated downstream of TSSs, at a distance less than 50 kb (Fig. 5 and Additional file 5). In keeping with this, analysis of their position in relation to TSSs revealed 30 sequences adjacent to and 4 ones residing within TSSs, as exemplified in Fig. 4a-f, and 22 sequences were located within introns while 2 ones at the 3′ end of genes (Table 1 and example in Fig. 4). Evaluating their integration distance from known genes, we found a statistically significant association of VL30 sequence integrations in the vicinity of genes encoding zinc finger proteins (ZFPs) (binomial raw *p*-value, *p* = 1.23 × 10^−10^), which contain the Krueppel-associated box (KRAB) (Additional file 6). Finally, we also found sequences located near regulatory genes such as PSF (*Sfpq*) (Fig. 4e), and several transcription factors (*Gtf2e2, Ap1m1, Crebzf*), kinases (*Cks2, Camkk2*), receptors (*Gpr113, Olfr1033, TCR-beta chain*), as well as genes involved in cell differentiation (*Tnks, Zfp568, Tdrd9, α*) (Table 1).

**Table 1:** VL30 elements closely located to mouse genes

Region	VL30	Associated gene, (distance to TSS)	Orientation^a^	Possible role
Adjacent to TSS	4qA1-2	Rbm12b (−4469)	+	Alternative promoter, Enhancer Or Effects in gene expression
	4qE2	Ube4b (−9958)	−	
	5qG2	Rhbdd2 (−6349)	+	
	6qA2	Naa38 (−3143)	+	
	7qA3-2	Eid2 (−8861)	+	
	8qA4	Gtf2e2 (−15672)	+	
	8qB33	Ap1m1 (−3642)	+	
	8qC3	Gm6531 (−12209)	+	
	8qD3	Tmco7 (−4073)	+	
	12qC1	Fbxo33 (−5398)	−	
	13qA5	Cks2 (−8731)	+	
	15qE1	Ndufa6 (−10978)	−	
	18qA1-2	Rbbp8 (−12595)	+	
	8qE1tr	Afg3l1 (−3684)	−	
	RLTR1C-chr6-1	Tmem168 (−3944)	−	
	RLTR1D-chr5-3	Camkk2 (−2560)	+	
	RLTR1C-chr4-1	Zmym1 (−2767)	+	
	RLTR1D-chr8-2	Tnks (−5185)	+	
	RLTR1D-chr7-5	Crebzf (−1957)	+	
	RLTR1D-chr4-6	Sfpq (−18784)	−	
	RLTR6_Mm-chr5-4	Gpr113 (−865)	−	
	RLTR6_Mm-chr6-2	Avl9 (−4915)	−	
	RLTR1C-chr4-2	Fam54b (−3954)	+	
	RLTR6_Mm-chr5-6	Mrfap1 (−2311)	+	
	RLTR1D-chr7-2	Ceacam5 (−4916)	−	
	RLTR6_Mm-chr7-2	Zfp568 (−4304)	+	
	RLTR6_Mm-chr11-2	Rpl23 (−2735)	+	
	RLTR1C-chr13-1	Mrpl32 (−4663)	−	
	RLTR1D-chr13-2	Ankdd1b (−3400)	−	
TSS within VL30	1qD	AK077157 (0)	−	Promoter
	2qD	Olfr1033 (0)	+	
	4qB3tr	Gm12505 (0)	−	
	18qA2	AK076984 (0)	−	
Intronic	1qA2	Sntg1 (+62021)	−	Alternative splicing region or disruption of transcription
	2qA3	Abi1 (+27853)	−	
	6qB1	TCR-beta chain (+20882)	+	
	6qG2	Slco1a6	−	
	7qA3	gm6902	−	
	12qA11	Slc7a15	−	
	12qF1	Tdrd9 (+47580)	+	
	12qa13	Gm4983	+	
	7qc3	A330076H08Rik	−	
	7qE2tr	Gm1966 (+26035)	−	
	7qE2-2tr	Neu3 (+9192)	−	
	RLTR1D-chr14-2	Dydc2 (+5144)	−	
	RLTR1D-chr1-12	Parp1 (+5484)	−	
	RLTR6_Mm-chr2-2	Ap4e1 (+13898)	+	
	RLTR6_Mm-chr2-3	Plk1s1 (+34325)	+	
	RLTR6_Mm-chr2-4	a (+15736)	+	
	RLTR1C-chr3-1	Slc7a12 (+4176)	−	
	RLTR6_Mm-chr5-9	N4bp2l2 (+1602)	+	
	RLTR6_Mm-chr8-2	Gsr (+11508)	+	
	RLTR1C-chr9-2	Rasgrf1 (+76260)	−	
	RLTR1D-chrX-2	Otc (+44070)	−	
	RLTR1D-chr14-2	Dydc2 (+5144)	−	
End of gene	RLTR6_Mm-chr11-1	Wdr92 (+22760)	+	Transcription termination
	RLTR1D-chr6-3	BC064078	+	

^a^+ same orientation to that of gene, − opposite orientation

### Phylogeny of VL30 and estimation of their integration time in the mouse genome

There is a relatively limited data on VL30 phylogeny concerning only a small subset of 18 sequences, which was obtained prior to mouse genome sequencing [5].

**Fig. 6:**
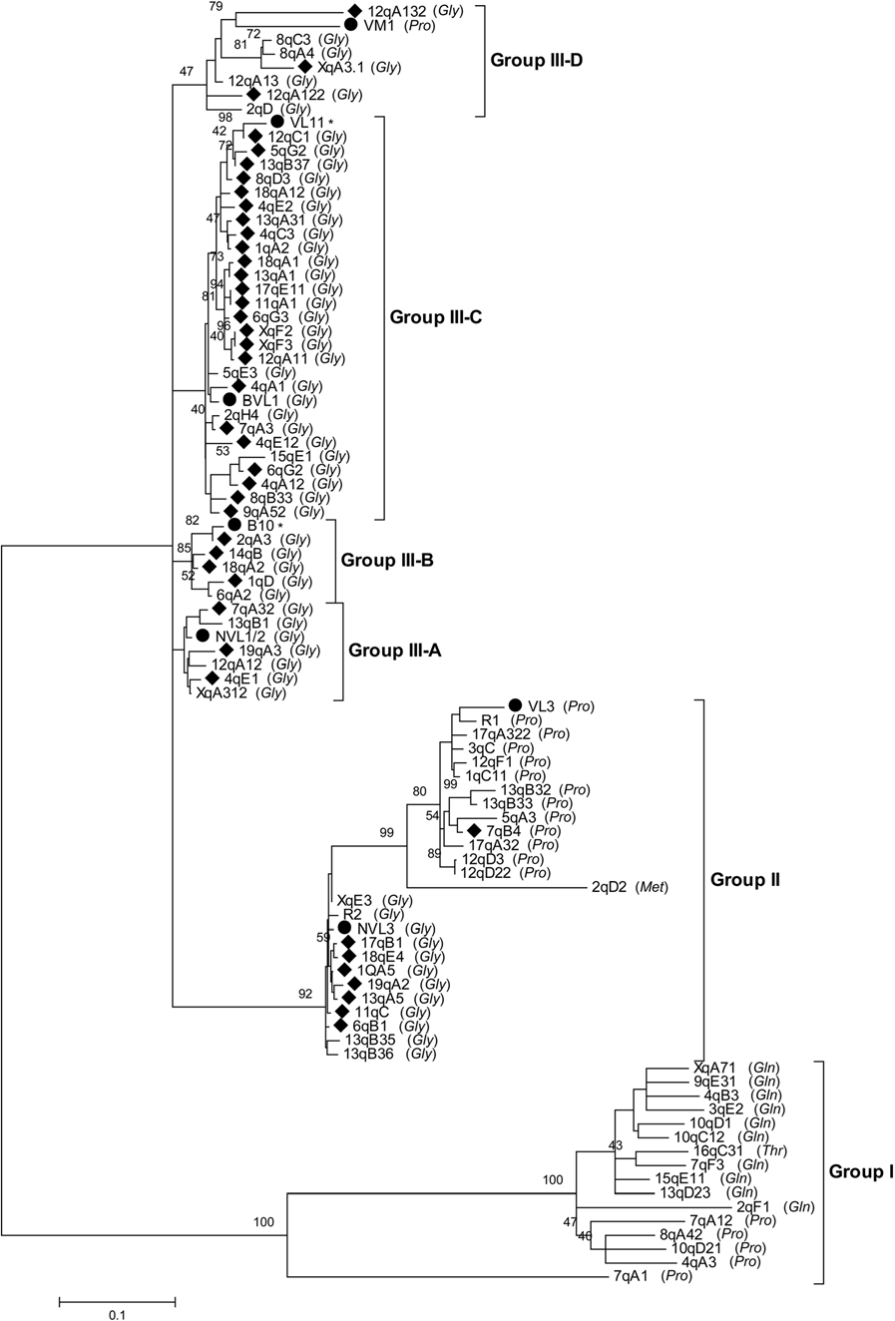
Molecular phylogenetic analysis of full-length VL30 LTRs by the Maximum Likelihood method. The bootstrap consensus tree representing the phylogeny of LTRs from 86 full-length and 6 known VL30s is presented (500 tests). The percentage of replicate trees in which the associated taxa clustered together is shown above the branches. The tree is drawn to scale, with branch lengths measured in the number of substitutions per site. Bootstrap replicates denote known elements or elements with identical 5′ and 3′ LTRs. The respective tRNA species used by each VL30 element is shown in parenthesis. Asterisk denotes solo LTR sequences

To obtain information on VL30 phylogeny, we attempted a relative analysis of VL30 sequences based on differences of their 5′ LTR, which mostly define the functional properties of each element. We avoided analysing truncated copies since they show extensive divergence in LTR length (truncated LTRs) and even absence of entire LTR regions. We used ClustalW2 algorithm [44] to perform multiple alignment between the 86 5′LTR of full-length elements and as reference the LTR sequences of seven known VL30s (VM1, VL11, BVL1, B10, NVL1/2, NVL3 and VL3 elements) [4, 5]. We generated a phylogenetic tree using the Maximun Likelihood method and Tamura-Nei model, for correction of multiple hits by taking into account the differences in substitution rate between nucleotides and the inequality of nucleotide frequencies. Based on the phylogenetic tree obtained (Fig. 6), we found that VL30 elements have diverged into three distinct groups. Group I was the oldest group comprising 16 elements, which were not closely related to the aforementioned reference sequences, forming a separate monophyletic group to the elements of group II and III. Group II contained 24 elements related to NVL3 and VL3 in two sub-groups; one highly related to NVL3 and a divergent one more related to VL3. Finally, Group III contained 46 elements divided in four sub-groups: III-A, III-B and III-C, which were highly similar while that of III-D was more divergent. This phylogenetic grouping can be confirmed by the tRNA species used by each element. Specifically, Group I is quite heterogeneous as 10, 5 and 1 members use Gln, Pro or Thr tRNA species, respectively. In contrast, Group II is divided into two distinct sub-groups containing either exclusively Gly (sub-group related to NVL3) or mainly Pro tRNAs (sub-group related to VL3). As regards Group III, with exception of one element, all others contain Gly tRNA (Fig. 6).

**Fig. 7:**
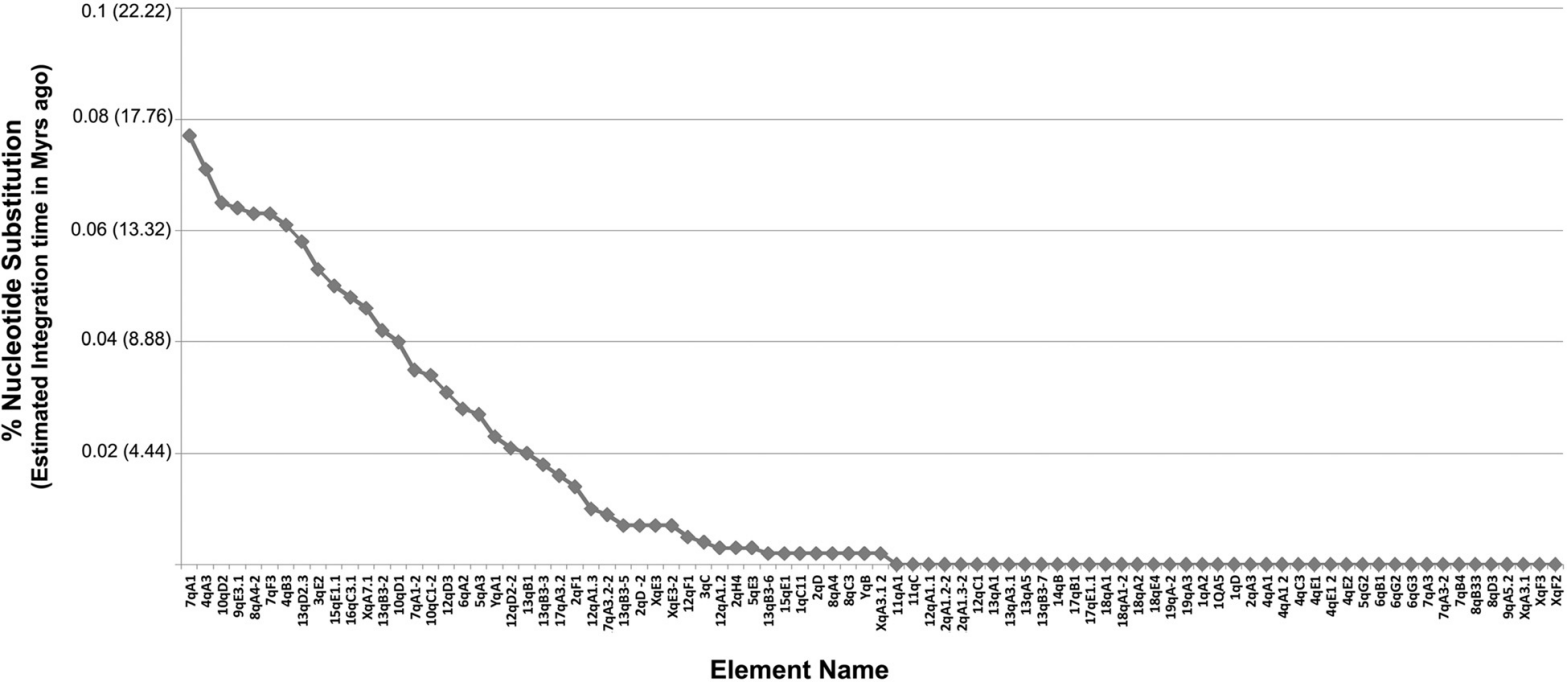
Estimation of divergence and time of integration for full-length VL30s. The graph represents the percentage (%) divergence of 5′ and 3′ LTRs, as found by BLAST querying, and the respective calculated time of integration, taking into account the mean mutation rate of the mouse genome (4.6 × 10^−9^ mutations per site per generation)

We next calculated the sequence divergence between paired 5′ and 3′ LTRs as previously described [45], in order to obtain evolutionary data for each individual element. We found that most VL30s shared highly related 5′ and 3′ LTRs pairs. In addition, using a mean nucleotide substitution rate of 4.6 × 10^−9^ [2], we also estimated LTR integration time [46]. We found that VL30s (Group I) emerged about 17.5 Myrs ago (Fig. 7) and most integrations date from 0.45 Myrs ago till today. Finally, 42 full-length elements shared identical 5′ and 3′ LTRs.

To confirm the evolutionary relationships analyzed, we combined the data of the two aforementioned analyses by highlighting with rhombus symbols the elements with identical 5′ and 3′ LTRs in the evolutionary tree obtained (Fig. 6). Indeed, Group I was the oldest one as its members shared no identical LTRs, and 7qA1, having the most divergent 5′ and 3′ LTRs, was the “oldest” VL30 element. Group II was the immediately younger subgroup comparing to Group I and contained 13 sequences (out of 21 total) highly similar to VL3 that shared identical 5′ and 3′ LTRs. Finally, Group III elements, although consisting of three highly related and one divergent subgroup, were the overall youngest group as their high majority, 34 out of 46 elements, had identical LTRs (Fig. 6). Collectively, based on the above dating analysis, Group I contains elements that are 17.5-3.1 Myrs old (from 7qA1 to 2qF1), Group II of 9.3 Myrs old up to date (from 13qB3.2 to 8 elements with identical LTRs) and the youngest Group III of 6.2 Myrs old up to date (from 6qA2 to 34 elements with identical LTRs).

## Discussion

The present study, based on the complete sequencing of the mouse genome and utilizing advanced bioinformatic tools, presents an *in silico* analysis of VL30s providing information about their structural characteristics, chromosomal distribution and evolution.

### Features of VL30 elements

Prior to this study, it was generally assumed that the mouse genome contained ~150–200 VL30 full-length elements [4], while our analysis revealed 372 total VL30 sequences. Specifically, we found 86 full-length elements, 49 truncated elements, and 237 solo LTR sequences. Accordingly, the total number of truncated and solo LTR sequences is 3.3-times more than those of full-length elements. This implies that both types of non-intact VL30 sequences rather derived after several mutation/recombination events during evolution. Hence, in contrast to previous assumptions, the mouse genome contains 86 full-length elements comprising the 23.1 % of the total VL30 sequences. 76 out of 86 full-length elements analysed had a hallmark of their genome integration, a conserved 4-bp TSD [4, 20]. LTRs were found to be quite heterogeneous, ranging from 436 to 681 bp. Thus, as VL30s are retrotransposition-competent [19], this finding might be explained by the occurrence of mutations during transcription and/or reverse transcription during retrotransposition, or by recombination events during evolution. Regarding the PBS signals, the large majority had Gly tRNA species specificity, while 17 and 10 elements had a Pro and Gln tRNA species (Fig. 1), respectively. Based on this, we may categorize full-length elements into three distinct groups according to the replication tRNA species: the prevalent group of Gly tRNA-positive elements and those of less frequent groups Pro- and Gln tRNA-positive elements. In addition, the different groups of tRNA species in association to conserved PPTs found (Fig. 1), shows the versatile nature of VL30s replication. Importantly, all internal sequences were characterized by the absence of an *env* gene while *gag* and *pol* genes had multiple stop codons, documenting that VL30s have no intact ORFs or code for very small peptides. Although VL30s are retrotransposition-competent these data emphasize that none of the full-length VL30s is an autonomous-LTR retrotransposon and their retrotransposition is mediated *in trans*-complementation, probably, by an endogenous MoMLV reverse transcriptase [19]. Strikingly, out of 135 total VL30 sequences, 86 full-length and 49 truncated, only 12 sequences - 3 full length and 9 truncated ones - had no PSF-binding motifs (Fig. 1 & Additional file 1). This large majority of PSF-positive sequences, irrespective of their full length or truncated state, document an almost universal property of VL30s, unique among retroelements, involved in mouse steroidogenesis and oncogenesis [39]. Finally, we were unable to locate or determine a specific or consensus Ψ signal for mouse VL30s, following comparison with that of rat VL30s. Though mouse VL30 elements undoubtedly have a strong but unknown yet Ψ signal, as their RNA can be encapsidated in C-type retroviruses [12]. Thus, we believe that the different Ψ signal in rat and mouse VL30 elements distinguishes evolutionarily these murine species.

### VL30s-related transcription factors and ESTs

It is well known that cellular transcription factors (TFs) interacting with specific DNA motifs located in 5′ LTRs regulate retroviral transcription [47]. We found that although the number of TF binding sites (TFBS) largely varied depending on the particular VL30 LTR, we identified 10,371 TFBS allocated to 53 distinct TFs, common to 70 % of 86 new full-length elements, and a total number of 14,880 TFBS from 197 TFs in all 5′ LTRs analyzed (Additional file 2). Notably, while some TFBS were present in almost all full-length elements such as those of HOMF and VTBP, others were extremely infrequent with 1–2 TFBS in only one or both LTRs (Additional file 2). In relation to the glucocorticoid responsive/related TFBS of GREF, they were present in all LTRs of full-length elements with 264 total binding sites, ranging from 1 to 7 sites per element. This particular finding justifies our previous observation on VL30 RNA induction through nuclear steroid/receptor complexes binding on GREF following estradiol, diethylstilbestrol, progesterone or dexamethasone treatment [8]. Significantly, we identified 52,986 total TFBSs in both truncated as well as solo LTR sequences that may be similarly involved in gene expression, as full-length VL30 LTRs. In support of this comes the finding that 99 distinct VL30 sequences had 592 VL30-associated ESTs (Additional file 3), implying their involvement in initiation or termination of RNA expression. In conclusion, VL30s: (a) bear a large number of common or unique TFBS, explaining the versatile VL30 RNA expression, which can be tissue-specific or up-regulated by various or pleiotropic stimuli, and (b) provide transcriptional initiation or termination sites, which may affect cellular gene expression.

### VL30s genomic distribution

The analysis of chromosomal distribution of VL30s shows that they are ubiquitous in mouse autosomal and sex chromosomes (Fig. 2) and integrated in regions of ~42 % GC content (Additional file 4), which is the exact GC average of the mouse genome [2]. Nevertheless, two lines of evidence support their preferential chromosomal integration documented by: (a) chromosomes 3, 7, 12, 13, 17 and X where the number of integrations is higher that than those expected (Fig. 3), and (b) a possible integration “hot-spot” mapped in 1.5 Mb of XqF2-F3, where 8 truncated VL30 elements were inserted (Fig. 4) probably as a result of serial unequal recombination events between loci of the X chromosome.

As concerns VL30 genomic distribution with respect to gene regions, we found that the large majority of 372 VL30 sequences were preferentially integrated near or in gene regions. In particular, 285 or 272 integrations were located upstream or downstream of TSSs, respectively, in a distance less than 500 Kb (Fig. 5). The significance of such integrations is that they may play an important role in the epigenetic regulation of gene expression. Specifically, first, VL30s affect nearby gene expression acting in a distance of up to 220 Kb [14], and contain many TFBS. Based on this, VL30 integrations at about of <20 Kb distance upstream of 29 known genes (Table 1) may play a *cis*-acting regulatory role in modulating their expression. Thus, VL30 LTRs integrated adjacent, upstream or downstream of gene Transcriptional Start Sites (TSS) (Fig. 5) may act as promoters or enhancers for a large number of genes (Table 1). Notably, VL30 members are retrotransposition-competent and their retrotransposition is induced up to ~90,000- or 420,000-times higher than the natural retrotransposition frequency by arsenic [23] or H_2_O_2_ [21], respectively. Conceivably, in these cases, the number of new integrations might lead to a dramatic change of the whole genome expression profile. Secondly, we found three full-length and one truncated VL30 element containing a TSS (Table 1) and their LTRs may serve as primary promoters for these genes. Thirdly, 22 integrations found inside introns and 2 ones at the 3′ end of genes (Table 1). Intronic integrations may affect mRNA splicing, as the presence of splicing donor sequences in some VL30s [4] is consistent with this concept. In reference to integrations at the 3′ end of genes (Table 1) we believe that they possibly act as transcription termination signals through VL30 polyA sequences since retrotransposon integrations lead to premature transcriptional termination even at a >12.5 Kb distance [48].

Finally, we identified a significant number of integrations near genes of the KRAB (Krueppel-associated Box containing) family (Additional file 6), known to participate in epigenetic silencing of endogenous retroviruses [49, 50]. Given that: (i) retrotransposons participate in or control regulatory networks in embryonic stem cells [51] or more premature developmental stages [52]; (ii) VL30s contain a large repertoire of TFBS (Additional file 2) and are competent of inducible [5, 8] and cell type-specific expression [6]; and (iii) Mouse VL30s inserted near genes affect their expression [14]; we suggest that the unique genomic distribution of VL30s near genes, such as those of the KRAB gene family, might ultimately lead to their selection as functional elements influencing epigenetic gene regulation and participating in regulatory networks.

### VL30 phylogeny

Based on the evolutionary tree created in the present study (Fig. 6) and on the calculation of the integration time for individual VL30s phylogeny (Fig. 7), we found that VL30s are divided into three evolutionary groups.

Our dating analysis is based on nucleotide substitution rate that creates divergence between 5′ and 3′ LTRs of individual elements. Even though we cannot exclude possible biases in date estimates (deamination biases for instance), we followed that method since it is the most widely accepted for LTR elements, has been used for HERV elements [45] and our data from intra-element data analysis come in agreement with their position in the phylogenetic tree. We document that Group I is the oldest one emerged ~17.5 Myrs ago, after the calculated rodent-primate divergence of ~41 Myrs ago [53] and this may explain why VL30s are rodent-specific, not existing in primates. Moreover, the element 7qA1 seems to be the archetype or “the VL30 eve” (Fig. 7). Group II and Group III elements comprised mostly of full-length elements, emerged 9.3 and 6.2 Myrs ago, respectively and are characterized by highly conserved LTRs (Fig. 6) and replication signals (Fig. 1). The elements of both groups are active or retrotransposition-competent, as 42 out of 86 full-length elements share identical 5′ and 3′ LTRs representing present-day integrations, and being possible candidates for the evolutionary youngest elements. VL30 grouping is further strengthened by the fact that while Group I elements are characterized by heterogeneity using 3 different tRNA species, Group II and Group III contained elements using Gly or Pro and Gly tRNA species, respectively (Fig. 6 and Additional file 1). We believe that the oldest elements probably diverged due to recombination/mutation events and upon evolutionary pressure underwent selection and fixation in the mouse genome. We cannot also exclude that these different PBS signals may indicate independently derived mutations from cross-species transmission events. Our data are also supported by previous findings that: (a) VL30s are highly polymorphic (at ~40 % of integrations) across 18 mouse strains [54]; (b) VL30 RNA is the only one (among LINEs, SINEs and IAP retroelements and DNA transposons) up-regulated by both histone hyperacetylation and DNA demethylation agents [7]; (c) overexpression of VL30 elements following DNA demethylation is associated with abnormal placental formation of *Mus musculus* × *Muscaroli* hybrids [55]; and (d) hydrogen peroxide induces VL30 retrotransposition of NVL3 element at ~42 % [21], the highest frequency ever measured in cultured mammalian cells. Taken together, our evolutionary data support that VL30 elements are highly active and rapidly expanding in the mouse genome.

Moreover, taking into account: (i) the recent expansion of VL30s in the mouse lineage (Fig. 2); (ii) the universal feature of VL30 RNAs to bind PSF (Fig. 1), leading to oncogenesis and steroidogenesis [15, 16]; and (iii) their possible role as insertion mutagens and inducers of cell death [24], we believe that PSF binding may act in some cases as a positive selection mechanism, leading to “tolerance” of VL30 integrations, since the deleterious effect of new retrotranspositions may be compensated for by the physiological action of VL30 RNA in steroidogenesis.

## Conclusion

This is the first extensive study on the LTR retrotransposon VL30s, which reveals that the mouse genome harbors precisely 372 total VL30 sequences characterized as 86 full-length, 49 truncated and 237 solo LTRs. The full-length VL30s are categorized into three distinct evolutionary groups. The finding that 42 out of 86 ones share identical LTRs and are marked by various genomic TSDs implies their potential to mobilize as non-autonomous retrotransposons, leading thus to a significant genome reorganization. A hallmark of VL30 genomic distribution is their preferential integration in 6 out 21 mouse chromosomes. At the transcriptional level, they can be diversely induced by various stimuli as they bear a very large number of TFBS also related to tissue-specificity. The most striking functional mark of VL30s, among other retroelements, is their involvement in steroidogenesis and oncogenesis as 91.2 % of both full length and truncated ones are PSF-positive. Finally, the large majority of the 372 sequences bearing LTR integrated near or in gene regions denotes their role in gene regulation, serving either as promoters/enhancers for many cellular genes or transcription termination signals through their polyA-signals. Retrotransposons, resembling the Janus double-faced Roman god, have a dual impact on the genome. Specifically, while new integrations of the retrotransposon copies might be mutagenic or deleterious leading to cell death their LTRs could be exploited by the cell for gene regulation, adaptation and general homeostasis upon environmental changes. Conclusively, VL30s, fulfilling both effects of retrotransposon action, might have been evolved as important mouse “LTR controlling elements” in parallel to those that Barbara McClintock stated for maize transposons [55].

## Methods

### Bioinformatics mining tools for identification of VL30 sequences

The UCSC genome browser (http://genome.ucsc.edu/) [30, 31] bioinformatic tools were used to screen manually the mouse genome (version mm9) for VL30 sequences. A combination of Repeat Masker analysis (http://www.repeatmasker.org) [32], BLAT querying [33] for VL30 consensus sequences from Repbase [34] and *in-silico* PCR for specific VL30s primers [35] was used to annotate VL30 sequences in their respective genomic regions. The LTR-finder tool (http://tlife.fudan.edu.cn/ltr_finder/) [36] was utilized to identify full-length VL30s. Sequences not corresponding to full-length VL30s were manually annotated as truncated VL30 elements or solo LTRs. The transfer of VL30 genomic coordinates between mouse genome version mm9 and the current one of mm10 was performed by the LiftOver tool of UCSC database.

The BLAST algorithm [56], in the NCBI database (http://blast.ncbi.nlm.nih.gov/Blast.cgi), was used to locate PSF binding motifs, (based on the two consensus sequences described in [15]) and the open reading frame (ORF)-finder [41] to search for potential ORFs in full-length VL30s. The analysis of full-length VL30 LTRs for transcription factor binding sites was performed using the Matinspector tool [40] in the Genomatix database (www.genomatix.de/matinspector.html). Retroviral Poly-Purine Tract (PPT) and PSF-binding motif consensus sequences for full-length elements were generated using the Weblogo software [57]. The GC-content of genomic regions [58], adjacent to full-length VL30s, was calculated in the Galaxy platform [59].

### Phylogenetic and nucleotide substitution analysis

The multiple alignment of VL30 5′ LTR regions was performed with the ClustalW2 algorithm [44] (http://www.ebi.ac.uk/Tools/msa/clustalw2/). The phylogeny of VL30s was inferred using the Maximum Likelihood method, based on the Tamura-Nei nucleotide substitution model [60], including in our analysis the LTRs of seven known elements (NVL1/2, NVL3, BVL1, VL3, VM1, VL11 and B10). The bootstrap test (500 replicates) was applied to validate phylogeny [61] and all evolutionary analyses were conducted by the molecular evolutionary genetics analysis 5 (MEGA5) software [62].

Nucleotide substitution analysis was performed according to a previous study [63] to calculate the divergence between paired 5′ and 3′ LTRs, as described for HERV retrotransposons [45]. Paired 5′ and 3′ LTR sequences were compared by BLAST alignment [56] to find differences in nucleotide sequence, as product of spontaneous mutation events during evolution. Based on the number of nucleotide substitutions between paired LTRs we calculated VL30s integration time according to an estimate of the mean mutation rate in the mouse genome, 4.6 × 10^−9^ mutations per base per year [2].

### Analysis of VL30s genomic distribution

The distribution of VL30s was depicted using the Ensembl Genome Browser [42] uploading their genomic coordinates. To study VL30 chromosomal distribution, chromosome sizes were obtained from summary tables in UCSC website and the fraction of each chromosome was calculated (by dividing the chromosome size to the total genome size). The expected number of element integrations per chromosome was calculated by multiplying the total number of elements with chromosome fraction, as done in a previous study for SVA elements [64]. The chi-squared test was used to analyze the chromosomal distribution of VL30 elements by comparing the observed with the expected number of VL30 elements, assuming a random insertion model.

The relative distribution of VL30s to Transcription Start Sites (TSS) of mouse genes was analyzed by the Genomic Regions Enrichment of Annotations Tool (GREAT) (great.stanford.edu) [43], with VL30 coordinates uploaded to UCSC Table Browser [65]. Briefly, GREAT algorithm determines the distance of a given genomic region to the nearest TSS found in a distance less than 1000 Kb. Then it categorizes elements based on the respective distance in groups (0–5 Kb, 5–50 Kb, 50–500 Kb and >500 Kb). Finally, it calculates of association between elements and previously annotated gene groups. The obtained association data were manually confirmed and further examined in the UCSC Genome Browser.

### Statistical analysis

STATISTICA (version 13) was used for statistical analysis. The chi-squared test was used to analyze chromosomal distribution of VL30 elements, based on the expected values from a random distribution model. VL30s and gene TSS association statistics were calculated in the GREAT platform [43]. GREAT performs a binomial test over genomic regions and the hypergeometric test over genes, so that a possible bias from the one test is compensated by the other test. Only the output regions with significant associations from both independent statistical tests were obtained.

## Additional files

Additional file 1: Genomic coordinates and structural features of full-length, truncated and solo-LTR VL30 sequences. The structural features of each individual full-length element after LTR-finder analysis are shown using the genome version mm10. The presence of PSF-binding motifs, with the percentage (%) similarity for each motif to consensus sequence is also shown for full-length and truncated elements. (XLS 79 kb) Additional file 2: Common and unique transcription factor binding sites in LTRs of full-length, truncated and solo LTR VL30 sequences. The Microsoft Excel file contains three sheets each one presenting all transcription factor binding sites of full-length, truncated and solo LTR VL30 sequences, respectively. (XLS 554 kb) Additional file 3: ESTs associated with VL30 elements. The Microsoft Excel file contains detailed information about the ESTs starting or terminating within distinct VL30 sequences. (XLS 84 kb) Additional file 4: GC-content adjacent to full-length VL30s integrations. The GC-content of 400 bp upstream and downstream of full-length VL30s was analyzed in the Galaxy platform. The graph shows the average GC-content in each chromosome. Error bars represent 95 % confidence intervals (+/- the standard deviation). (JPG 483 kb) Additional file 5: VL30 elements integrated nearby mouse genes. The table provides information about all VL30 elements integrated in the vicinity of mouse genes and their relative distance to transcription start sites (TSS). (PDF 254 kb) Additional file 6: VL30 elements associated with Krueppel-associated box (KRAB) zinc finger proteins. The table provides information about all VL30 elements associated with KRAB zinc finger proteins and their relative distance to TSS. (PDF 10 kb)

## Abbreviations

EST: expressed sequence tag
KRAB: Krueppel-associated box
LTR: Long Terminal Repeat
PSF: poly-pyrimidine tract-binding protein-associated splicing factor
TFBS: transcription factor binding site
TSD: Target Site Duplication
TSS: transcription start site
VL30s: viral like 30 elements
