# Supplementary figures and images for: Genomic analysis of mouse VL30 retrotransposons
Source: Mob DNA. 2016 May 6;7:10. doi: 10.1186/s13100-016-0066-8 (PMC4859993; doi:10.1186/s13100-016-0066-8)

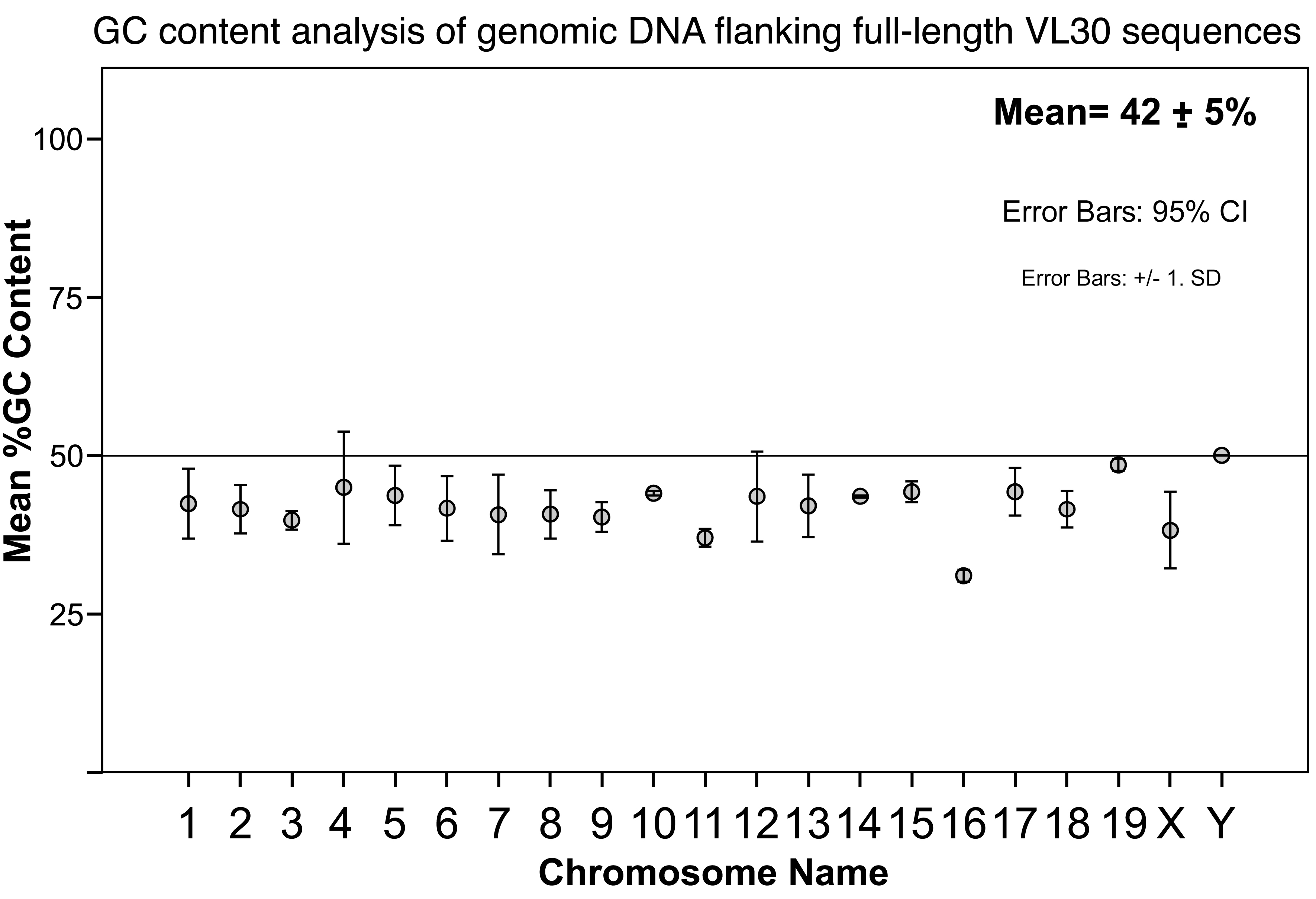

Supplement: Additional file 4: — GC-content adjacent to full-length VL30s integrations. The GC-content of 400 bp upstream and downstream of full-length VL30s was analyzed in the Galaxy platform. The graph shows the average GC-content in each chromosome. Error bars represent 95 % confidence intervals (+/- the standard deviation). (JPG 483 kb) [file 13100_2016_66_MOESM4_ESM.jpg]
