# Supplementary material for: Genomic analysis of mouse VL30 retrotransposons
Source: Mob DNA. 2016 May 6;7:10. doi: 10.1186/s13100-016-0066-8 (PMC4859993; doi:10.1186/s13100-016-0066-8)
Supplement: Additional file 5: — VL30 elements integrated nearby mouse genes. The table provides information about all VL30 elements integrated in the vicinity of mouse genes and their relative distance to transcription start sites (TSS). (PDF 254 kb) [file 13100_2016_66_MOESM5_ESM.pdf]

VL30 name	Gene name/Distance to TSS
1qA2	Sntg1 (+62021)
1qA5	Lmbrd1 (+388746), Bai3 (+812423)
1qC1.1	Asnsd1 (-342063), Stk17b (+90453)
1qD	Alppl2 (+11836), Dis3l2 (+374288)
2qA3	Abi1 (+27853), Pdss1 (+116865)
2qD-2	Zfp804a (-62350)
2qD	Olfr1033 (+335)
2qF1	Tgm3 (-63127), Stk35 (+148730)
2qH4	Gm14420 (-211075), AK078446 (-47223)
3qC	NONE
3qE2	Otol1 (-83575), 1110032A04Rik (-64142)
4qA1	Car8 (-156331), Rab2a (-140272)
4qA1-2	Rbm12b (-4469)
4qA3	Manea (-702032)
4qB3	Mup5 (-62217), Mup20 (+156720)
4qC3	4930473A06Rik (+13866)
4qE1	Gm13051 (-45787), RP23-282C23.3 (+52398)
4qE1-2	Gm13235 (-32771), Zfp600 (+189055)
4qE2	Ube4b (-9958), Rbp7 (+18379)
5qA3	Magi2 (-568586), Gnai1 (-298047)
5qE3	Gm6205 (-129549), AA792892 (+141458)
5qG2	Ccl24 (-53262), Rhbdd2 (-6349)
6qA2	Naa38 (-3143)
6qB1	H4 (-28068), TCR-beta chain (+20882)
6qG2	Gm6614 (-161345), Slco1a6 (+16059)
6qG3	Tmtc1 (-193333), Ipo8 (+193782)
7qA1	Vmn2r52 (-73798), Vmn1r66 (+25267)
7qA1-2	Zscan4d (+24980), Zscan4c (+135423)
7qA3	Vmn1r109 (+13803), Vmn1r159 (+68105)
7qA3-2	Eid2 (-8861), Dyrk1b (+79537)
7qB4	Mrgprb3 (-97462), Zdhhc13 (-47740)
7qF3	4933402N03Rik (-17158), 5430419D17Rik (-10961)
8qA4	Gtf2e2 (-15672), Gsr (+63004)
8qA4-2	Msr1 (+150541), Tusc3 (+508570)
8qB33	Ap1m1 (-3642)

8qC3	Zfp791 (-16620), Gm6531 (-12209)
8qD3	Tmco7 (-4073)
9qA5-2	Gm5617 (+25888), Nnmt (+83844)
9qE31	Tbc1d2b (-266264), Zic1 (+828766)
XqA3-1-2	Gm14351 (-253364), Gm2799 (-51916)
XqA3-1	Gm2933 (-141676), Gm14374 (+112580)
XqA7-1	Slitrk2 (-698101), Ctag2 (+903573)
XqE3	Vmn2r121 (-146875)
XqE3-2	Vmn2r121 (-816915)
XqF2	Dcx (-342862), Alg13 (-41885)
XqF3	Alas2 (-59918), Tmem29 (-46362)
YqA1	NONE
YqB	NONE
10qC12	D10Jhu81e (+46187), Icosl (+54213)
10qD1	Ppfia2 (-311445), Ccdc59 (+317415)
10qD2-1	Atxn7l3b (-742726)
11qA1	Ankrd36 (-14253), Ccdc117 (-13214)
11qC	Dhx40 (-6409), Ypel2 (+179693)
12qA1-1	Pum2 (-88731), Slc7a15 (-45983)
12qA1-2-2	5730507C01Rik (-641417), Hpcal1 (+182279)
12qA1-2	5730507C01Rik (+604420)
12qA1-3-2	NONE
12qA1-3	Gm4983 (+14194)
12qC1	Fbxo33 (-5398)
12qD2-2	Oog1 (-50760), Gm2022 (+10909)
12qD3	Gm10436 (+18419), Oog1 (+208972)
12qF1	Aspg (-87544), BC048943 (-38284)
13qA1	Tbce (+16960), B3galnt2 (+67966)
13qA3-1	Hist1h2bn (+11370), Hist1h1b (+15061)
13qA5	Shc3 (-69417), Cks2 (-8731)
13qB1	Nsd1 (-22938), Fgfr4 (+34026)
13qB3-7	Gm3604 (+88879), Zfp808 (+164405)
13qB3-2	Gm10324 (+271944), Zfp640 (+312837)
13qB3-3	Zfp640 (-71404), Uqcrb (+135712)
13qB3-5	Mtrr (+346485), BC048507 (+410239)
13qB3-6	Mtrr (+283492), BC048507 (+473232)

13qD2-3	Mrps30 (+332422), Hcn1 (+452510)
14qB	Msmb (-24098), Oxnad1 (+32197)
15qE1-1	A4galt (-18016), Arfgap3 (+80457)
15qE1	Ndufa6 (-10978), Cyp2d22 (+14991)
16qC3-1	Cxadr (-583266), Usp25 (+704356)
17qA3-2	Vmn2r97 (-14266), Vmn2r96 (+318329)
17qA3-2-2	Vmn2r103 (+119648), Vmn2r104 (+155194)
17qB1	Pou5f1 (-25353), H2-Q10 (+10597)
17qE1-1	Twsg1 (-66610), Ankrd12 (+59249)
18qA1	Rab18 (-98722), 4921524L21Rik (+62850)
18qA1-2	Rbbp8 (-12595), Gata6 (+751248)
18qA2	Celf4 (-251607)
18qE4	Tmx3 (-672104), Dok6 (-68914)
19qA2	BC021614 (-4581)
19qA3	Ighmbp2 (+56760)
1Qc1.1tr	Asnsd1 (-35506), Stk17b (+397010)
1qE2.1tr	Cdh19 (-162359), Dsel (+725187)
1qE6tr	Cr2 (-166473)
2qC3tr	Hnrnpa3 (-18735), Mtx2 (+814712)
3qA1tr	Cyp7b1 (-47568), Armc1 (+872159)
3qA3tr	NONE
3qE1tr	Nmd3 (-68751), B3galnt1 (-54443)
3qE3tr	Golim4 (+141030), Serpini1 (+258268)
4qB3tr	Rad23b (+70667), Klf4 (+111766)
4qD21tr	Dmap1 (-69523), Klf17 (+13918)
6qB23tr	Krba1 (-160764), Zfp746 (-148229)
6qB3tr	Snca (-62174), Mmrn1 (-53147)
7qA2tr	Ceacam5 (-172706), Psg29 (+337069)
7qB1tr	Abpe (+458370)
7qB1-2tr	LOC545947 (+790358)
7qCtr	Ndn (-383974)
7qE2tr	Neu3 (+9192), Olfr521 (+52061)
7qE2.2tr	Gvin1 (-403270), Gm1966 (+26035)
8qE1tr	Afg3l1 (-3684)
9qA3tr	Zfp809 (+2382), Zfp599 (+31810)

9qDtr	Hcrtr2 (-103766), Fam83b (+118499)
XqF3-2tr	Gm10439 (-275981), Ott (+230305)
XqA11tr	Cybb (-14367), Gm5132 (+88700)
XqC1tr	4930415L06Rik (-158966)
XqE1tr	Cpxcr1 (-621398), 4930524E20Rik (+919964)
XqF2tr	Ott (+53785)
XqF2-2tr	Ott (-720017), Ott (+375305)
XqF2-3tr	Ott (-553285), Ott (+542037)
XqF3tr	Ott (-150583), Ott (+944739)
XqF3.2tr	Gm10439 (-442883), Ott (+63403)
XqF3-3tr	Gm10439 (-93213), Ott (+413073)
XqF3-4tr	Gm15097 (-134158), Gm10439 (+55745)
XqF3-5tr	Gm5647 (-135860), Gm16390 (+175077)
10qD2tr	Kcnc2 (+281269), Atxn7l3b (+376634)
10qD3tr	Olfr824 (-17959), Olfr825 (+18310)
11qCtr	Pex12 (+41962), Slfn3 (+65685)
12qD2tr	Oog1 (-46004), Gm8300 (+42368)
13qB3tr	Cts3 (-517722), Zfp808 (-42041)
13qB3.2tr	BC048507 (+271424), Mtrr (+485300)
13qC3tr	NONE
14qA3tr	Ube2e2 (-498464), Gm5458 (+197204)
14qE23tr	Trim52 (+349413)
15qA1tr	2410089E03Rik (+109399), Nipbl (+165958)
16qB1tr	2310042E22Rik (+70696), Chrd (+350121)
16qC2tr	Speer2 (+7940)
17qA1tr	Slc22a2 (+10891), Slc22a1 (+80758)
17qA32tr	Zfp53 (-12824), Zfp51 (+25812)
18qA13tr	Usp14 (-22955), Rock1 (+128688)
18qE1tr	Txnl1 (-8492), Wdr7 (-7844)
RLTR1C-chr1-1	Rgs13 (-41684), Rgs1 (+30048)
RLTR1D-chr1-1	Mrpl15 (-14622), Lypla1 (-7545)
RLTR1D-chr1-2	Pgap1 (-25839), Ankrd44 (+342866)
RLTR6_Mm-chr1-1	Scg2 (+187053), Kcne4 (+436088)
RLTR1D-chr1-3	Gin1 (+25745), Pam (+299708)
RLTR1D-chr1-4	NONE
RLTR1D-chr1-5	Serpinb11 (-42732), Serpinb3c (-41211)
RLTR6_Mm-chr1-2	Cdh19 (-808715), Dsel (+78831)

RLTR1D-chr1-6	NONE
RLTR1D-chr1-7	Cd55 (-10917), Zp3r (+155949)
RLTR1D-chr1-8	Fam5c (+692485)
RLTR6_Mm-chr1-3	NONE
RLTR1D-chr1-9	Cacna1e (+125339), Glul (+700636)
RLTR1D-chr1-10	Dpt (-263198), Atp1b1 (-75179)
RLTR1D-chr1-11	Pbx1 (+331881), Lmx1a (+410819)
RLTR6_Mm-chr1-4	Pbx1 (-169048), Nuf2 (+930158)
RLTR6_Mm-chr1-5	Ufc1 (-20303), Dedd (-13860)
RLTR1D-chr1-12	Lin9 (-66875), Parp1 (+5484)
RLTR1D-chr10-1	Moxd1 (-65213), Stx7 (+8987)
RLTR1D-chr10-2	L3mbtl3 (-363774), Arhgap18 (-33553)
RLTR6_Mm-chr10-1	Sult3a1 (-193972), Clvs2 (-39150)
RLTR6_Mm-chr10-2	Frk (+415961)
RLTR1C-chr10-1	Gpr6 (-225081), Fig4 (+6576)
RLTR6_Mm-chr10-3	Msl3l2 (-200798)
RLTR1C-chr10-2	Aifm2 (-14964), Tysnd1 (+10171)
RLTR6_Mm-chr10-4	Anks1b (-27988), Uhrf1bp1l (+100530)
RLTR1C-chr10-3	Btg1 (-550662), Eea1 (+125676)
RLTR1D-chr10-3	Syt1 (-302050), Nav3 (+687550)
RLTR1D-chr10-4	Cpm (-178260), Cpsf6 (-74267)
RLTR6_Mm-chr11-1	C1d (-22965), Wdr92 (+22760)
RLTR1D-chr11-1	Gapdh (+614437)
RLTR1D-chr11-2	Dppa1 (-65535), Timd2 (+12295)
RLTR1D-chr11-3	Dnahc9 (-240447), Shisa6 (+117128)
RLTR6_Mm-chr11-2	Rpl23 (-2735)
RLTR1D-chr11-4	Ptchd3 (+133225)
RLTR1D-chr12-1	Rdh14 (+347220), Kcns3 (+412373)
RLTR1C-chr12-1	5730507C01Rik (-429395), Hpcal1 (+394301)
RLTR1C-chr12-2	5730507C01Rik (+182737)
RLTR6_Mm-chr12-1	5730507C01Rik (+280393)
RLTR1C-chr12-3	5730507C01Rik (+281906)
RLTR6_Mm-chr12-2	5730507C01Rik (+307502)
RLTR1D-chr12-2	Asap2 (-197686), Gm4983 (+298976)
RLTR1C-chr12-4	Ywhaq (-709628)
RLTR1C-chr12-6	NONE

RLTR1C-chr12-5	NONE
RLTR1C-chr12-7	Taf1b (-349488), Gm4983 (-51824)
RLTR6_Mm-chr12-3	Gm4983 (-259744), Taf1b (-141568)
RLTR1D-chr12-3	Foxg1 (-391846)
RLTR6_Mm-chr12-4	Gm10436 (+90157), Oog1 (+137234)
RLTR6_Mm-chr12-5	Gm10436 (+82027), Oog1 (+145364)
RLTR1D-chr12-4	Dio2 (+143099)
RLTR1C-chr13-1	Mrpl32 (-4663), Psma2 (+4458)
RLTR6_Mm-chr13-1	5033411D12Rik (+8745)
RLTR6_Mm-chr13-2	Vmn1r205 (+19652), Vmn1r203 (+49227)
RLTR1D-chr13-1	Gcm2 (-36980), Elovl2 (+73435)
RLTR6_Mm-chr13-3	BC048507 (+7484), Mtrr (+749240)
RLTR6_Mm-chr13-4	Ccnh (-150705), Tmem161b (+816476)
RLTR1C-chr13-2	Homer1 (-95352), Papd4 (-16860)
RLTR6_Mm-chr13-5	Arsb (-9260), Dmgdh (+87983)
RLTR1D-chr13-2	Ankdd1b (-3400)
RLTR1D-chr13-3	Fam169a (-19945), 1700029F12Rik (-12980)
RLTR1D-chr13-4	Ipo11 (+23793)
RLTR1D-chr14-1	4930452B06Rik (-834936)
RLTR6_Mm-chr14-1	Sntn (-72846), Synpr (+313250)
RLTR1C-chr14-1	Asb14 (-105574), Dnahc12 (+18001)
RLTR6_Mm-chr14-2	Opn4 (-57983), Wapal (-15803)
RLTR6_Mm-chr14-3	Ghitm (-982337)
RLTR1D-chr14-2	5730469M10Rik (-50155), Dydc2 (+5144)
RLTR1D-chr14-3	Siah3 (-42503), Zc3h13 (+129106)
RLTR1D-chr14-4	NONE
RLTR6_Mm-chr14-4	NONE
RLTR6_Mm-chr14-5	NONE
RLTR1D-chr14-5	4921530L21Rik (-486451)
RLTR1D-chr14-6	Klf12 (-475425), 1700110M21Rik (+574847)
RLTR1C-chr14-2	Stk24 (-23187), Slc15a1 (+102837)
RLTR1D-chr15-1	Cdh10 (+571671)
RLTR1D-chr15-2	Cdh18 (-273732), Gm5803 (-

	47747)
RLTR6_Mm-chr15-1	Dnahc5 (+776482)
RLTR1D-chr15-3	Ctnnd2 (-671434)
RLTR1D-chr15-4	Cyp2d34 (-54159), Cyp2d37-ps (+15433)
RLTR6_Mm-chr16-1	Rbfox1 (-727207)
RLTR1C-chr16-1	Ube2v2 (-25518), Mcm4 (+17364)
RLTR6_Mm-chr16-2	2510002D24Rik (+14323), Mrpl40 (+25734)
RLTR1D-chr16-1	AI480653 (+81030), Fam43a (+400679)
RLTR1D-chr16-2	Tfrc (-71574), Zdhhc19 (+41041)
RLTR1C-chr16-2	Casr (-19122), Cd86 (+84821)
RLTR1C-chr16-3	Zbtb20 (-805346), Gap43 (-101287)
RLTR1D-chr16-3	Alcam (+197556), Cblb (+223892)
RLTR6_Mm-chr16-3	NONE
RLTR6_Mm-chr16-4	Krtap16-7 (-9191), Krtap6-2 (+7146)
RLTR6_Mm-chr17-1	Chd1 (-112011), Prdm9 (-29625)
RLTR1C-chr17-1	Rgmb (-901234), Zfp960 (-336283)
RLTR6_Mm-chr17-3	Zfp960 (-119120)
RLTR1D-chr17-1	Vmn2r98 (-97500), Vmn2r97 (+41671)
RLTR6_Mm-chr17-4	Vmn2r106 (-37277), Vmn2r107 (-22718)
RLTR6_Mm-chr17-5	Zfp229 (+41122), Zfp820 (+70913)
RLTR1D-chr17-2	4930539E08Rik (+25224), Pnpla1 (+31689)
RLTR1C-chr17-2	Pknox1 (-30090), Ndufv3 (+14568)
RLTR1C-chr17-3	March2 (-12327), Rab11b (+29489)
RLTR1D-chr17-3	H2-M10.6 (-12158), H2-M10.5 (+27107)
RLTR1D-chr17-4	Pla2g7 (-42558), Mep1a (-23120)
RLTR1C-chr17-4	NONE
RLTR6_Mm-chr17-2	Fert2 (-370729), Fbxl17 (-24709)
RLTR1D-chr17-5	Ddx11 (+55660), 1110012J17Rik (+270570)
RLTR1D-chr17-6	Kcnk12 (-107234), Msh6 (-69822)
RLTR6_Mm-chr18-1	Mpp7 (-236450), Wac (-5884)
RLTR1D-chr18-1	Thoc1 (-49359), Colec12 (+201173)
RLTR1D-chr18-2	Zfp191 (-50605), Ino80c (+50443)

RLTR1D-chr18-3	NONE
RLTR6_Mm-chr18-2	Wnt8a (+15039), Nme5 (+21739)
RLTR1D-chr18-4	Dtwd2 (+9134), Pol (+354868)
RLTR1D-chr18-5	Myo5b (-24775), Ccdc11 (+134744)
RLTR6_Mm-chr19-1	Gstp2 (-3129)
RLTR6_Mm-chr19-2	AB056442 (-123143), Gm5631 (+42812)
RLTR6_Mm-chr19-3	Foxd4 (-6916), Cbwd1 (+53391)
RLTR1D-chr2-1	Slc4a10 (-45909), Tbr1 (+196178)
RLTR6_Mm-chr2-1	Ttc30a1 (-323310), Pde11a (+33376)
RLTR1D-chr2-2	Zfp804a (-761417), Nup35 (+653429)
RLTR1D-chr2-3	Olfr1024 (+2997), Olfr1023 (+15253)
RLTR1D-chr2-4	Olfr1257 (-21094), Olfr48 (-14763)
RLTR6_Mm-chr2-2	Blvra (-48048), Ap4e1 (+13898)
RLTR6_Mm-chr2-3	Xrn2 (-122846), Plk1s1 (+34325)
RLTR6_Mm-chr2-4	a (+15736), Ahcy (+45191)
RLTR6_Mm-chr2-5	Mocs3 (+7391), Kcng1 (+31318)
RLTR6_Mm-chr3-1	Il7 (-841563), Stmn2 (-54537)
RLTR1D-chr3-1	Zbtb10 (-239942), Tpd52 (-46571)
RLTR1C-chr3-1	Lrrcc1 (-48913), Slc7a12 (+4176)
RLTR6_Mm-chr3-2	Cyp7b1 (-581148), Armc1 (+338579)
RLTR1D-chr3-2	Hltf (+31700), Gyg (+65484)
RLTR1C-chr3-2	Gyg (-8631), Cpa3 (+78454)
RLTR1C-chr3-3	Fat4 (-349726), Ankrd50 (-52398)
RLTR1C-chr3-4	Fat4 (-285996), Ankrd50 (-116128)
RLTR6_Mm-chr3-3	Pcdh10 (+857618)
RLTR1D-chr3-3	Vmn2r2 (-63765), Vmn2r3 (+86172)
RLTR1D-chr3-4	Kcnab1 (-25555), AK035994 (+134865)
RLTR1C-chr3-5	B3galnt1 (-99674), Nmd3 (-23520)
RLTR1D-chr3-5	Sis (+89910)
RLTR1C-chr3-6	Tlr2 (-42669), D930015E06Rik (+155884)
RLTR1D-chr3-6	Pglyrp4 (+248843)
RLTR1D-chr3-7	Pglyrp3 (-53190)
RLTR1D-chr3-8	Fcgr1 (-78772), BC107364 (+79677)
RLTR1D-chr3-9	Prmt6 (-604701)
RLTR6_Mm-chr3-4	St6galnac5 (+22987), Pigk (+245120)
RLTR1D-chr3-10	Slc44a5 (+67009), Lhx8

	(+290115)
RLTR1D-chr3-11	Negr1 (-621265), Lrriq3 (+847095)
RLTR6_Mm-chr4-1	Mms22l (-646611)
RLTR6_Mm-chr4-2	Olfr275 (-141955), 4930547C10Rik (+87170)
RLTR1D-chr4-1	3110001D03Rik (+71543), Kdm4c (+954864)
RLTR1D-chr4-2	NONE
RLTR1D-chr4-3	B020004J07Rik (-44484), C130073F10Rik (+5283)
RLTR1D-chr4-4	Pde4b (-105063), Gm12789 (+162839)
RLTR1D-chr4-5	0610037L13Rik (+16053), Cpt2 (+17637)
RLTR6_Mm-chr4-3	Bend5 (-247019), Agbl4 (+770196)
RLTR1D-chr4-6	Zmym4 (-34594), Sfpq (-18784)
RLTR1C-chr4-1	Zmym1 (-2767)
RLTR1C-chr4-2	Fam54b (-3954)
RLTR1D-chr4-7	Prdm2 (-32308), Pdpn (+54490)
RLTR6_Mm-chr4-4	Gm13101 (-8888), Pramef17 (+18531)
RLTR6_Mm-chr4-5	Gm13101 (-15015), Pramef17 (+12404)
RLTR6_Mm-chr5-1	4921511H03Rik (-384555)
RLTR6_Mm-chr5-2	Abcb1b (-43599), Abcb1a (+94456)
RLTR6_Mm-chr5-3	Galnt11 (+17764), Mll3 (+258126)
RLTR6_Mm-chr5-4	Gpr113 (-865)
RLTR6_Mm-chr5-5	Ywhah (-64491), Depdc5 (+90604)
RLTR6_Mm-chr5-6	Mrfap1 (-2311)
RLTR6_Mm-chr5-7	NONE
RLTR1C-chr5-1	G6pd2 (+394976), Arap2 (+562358)
RLTR1D-chr5-1	Amtn (-29059), Prol1 (+29737)
RLTR1D-chr5-2	Gbp4 (-39979), Mpa2l (+114168)
RLTR6_Mm-chr5-8	Tmem119 (-12021), Selplg (+18128)
RLTR1C-chr5-2	Aldh2 (-19573), 9330129D05Rik (+5541)
RLTR1D-chr5-3	Camkk2 (-2560)
RLTR6_Mm-chr5-9	Snx8 (-23756), Eif3b (-6302)
RLTR1D-chr5-4	N4bp2l1 (-69485), N4bp2l2 (+1602)
RLTR1D-chr6-1	Hepacam2 (+256928)
RLTR1C-chr6-1	Tmem168 (-3944)
RLTR6_Mm-chr6-1	Mpp6 (-91337), Npy (+196175)
RLTR6_Mm-chr6-2	Avl9 (-4915)
RLTR1D-chr6-2	Vmn1r19 (+29361), Ppm1k (+101601)
RLTR1C-chr6-2	AW146020 (-432087)

RLTR1D-chr6-3	Klrb1f (-39466), Clec2g (+72054)
RLTR6_Mm-chr6-3	Prh1 (-140998), Gm8882 (-64709)
RLTR6_Mm-chr6-4	Bicd1 (-48918), 2810474O19Rik (+50554)
RLTR6_Mm-chr6-5	Bicd1 (-17054), 2810474O19Rik (+82418)
RLTR1D-chr7-1	Aurkc (-3408)
RLTR1D-chr7-2	Ceacam5 (-4916)
RLTR6_Mm-chr7-1	Nlrp5 (-78167), Nlrp4e (+6538)
RLTR6_Mm-chr7-2	Zfp568 (-4304)
RLTR6_Mm-chr7-3	Hmg1l1 (-125781), Vmn2r58 (-22274)
RLTR1D-chr7-3	Zfp936 (-116385), Gm9268 (+42405)
RLTR6_Mm-chr7-4	Luzp2 (-862493)
RLTR6_Mm-chr7-5	Luzp2 (-414878)
RLTR1D-chr7-4	Ndn (-862600)
RLTR6_Mm-chr7-6	Olfr294 (+10810), Olfr295 (+20556)
RLTR1D-chr7-5	Crebzf (-1957)
RLTR1D-chr7-6	Trim12a (-6461), 9230105E10Rik (+31402)
RLTR6_Mm-chr7-7	Olfr651 (-39278), Trim30d (-5757)
RLTR1C-chr7-1	2310008H09Rik (-6862), Gprc5b (+132351)
RLTR1D-chr7-7	Acsm2 (-16571), Acsm5 (+18872)
RLTR1D-chr7-8	Shank2 (-297940), Dhcr7 (+54413)
RLTR6_Mm-chr8-1	Xkr5 (-19874), Defb40 (+7269)
RLTR1D-chr8-1	Zfp703 (-796580), Thap1 (+22587)
RLTR6_Mm-chr8-2	Gtf2e2 (-67168), Gsr (+11508)
RLTR1D-chr8-2	Ppp1r3b (-404866), Tnks (-5185)
RLTR6_Mm-chr8-3	Tusc3 (+209939), Msr1 (+449172)
RLTR1D-chr8-3	Msr1 (-430699), Fgf20 (+213576)
RLTR1D-chr8-4	Inpp4b (+177126), Il15 (+510218)
RLTR1D-chr8-5	Ucp1 (+16128), Elmod2 (+26010)
RLTR1D-chr8-6	1700008F21Rik (+48012)
RLTR1D-chr8-7	1700008F21Rik (+49997)
RLTR1D-chr9-1	Ddi1 (-126775), Dync2h1 (+783908)
RLTR1C-chr9-1	Ddi1 (-471530), Dync2h1 (+439153)
RLTR1D-chr9-2	Pgr (-125891), Trpc6 (+229800)
RLTR1D-chr9-3	Eepd1 (+207169)
RLTR1D-chr9-4	Tbcel (-306457), Grik4 (+165688)
RLTR6_Mm-chr9-1	Grik4 (-47120), Arhgef12 (+114227)
RLTR1D-chr9-5	Commd4 (-41376), Trcg1 (-36881)
RLTR6_Mm-chr9-2	Impg1 (-104826)

RLTR1C-chr9-2	Ctsh (-68232), Rasgrf1 (+76260)
RLTR1D-chr9-6	Plscr1 (-5724), Zic4 (+875498)
RLTR1C-chr9-3	Trf (-22242), 1300017J02Rik (+35769)
RLTR1D-chr9-7	Fbxw21 (+12278), Plxnb1 (+54308)
RLTR1D-chrX-1	Mycs (-163032), Gm14374 (-36770)
RLTR1D-chrX-2	Tspan7 (-188687), Otc (+44070)
RLTR1D-chrX-3	Dusp21 (-319406), Fundc1 (-254167)
RLTR1D-chrX-4	1700023I07Rik (-450159), Slc6a14 (+296537)
RLTR6_Mm-chrX-1	Gm4987 (+7740), Actrt1 (+119599)
RLTR6_Mm-chrX-2	Gm6760 (+391871)
RLTR1D-chrX-5	Msn (-117689), Las1l (-21397)
RLTR6_Mm-chrX-3	Fgf16 (-338091), Magee1 (+305990)
RLTR1D-chrX-6	Nap1l3 (+219823)
RLTR6_Mm-chrX-4	Vmn2r121 (-149986)
RLTR1D-chrX-7	4921511C20Rik (+11824)
RLTR1D-chrX-8	2900062L11Rik (-21506), Bhlhb9 (+86463)
RLTR1D-chrX-9	Kir3dl1 (-28212), Kir3dl2 (-20691)
RLTR1C-chrX-1	Hsd17b10 (-17728), Huwe1 (+180886)
RLTR1D-chrY-1	Rbmy1a1 (+254707)
