# Supplementary material for: Genomic analysis of mouse VL30 retrotransposons
Source: Mob DNA. 2016 May 6;7:10. doi: 10.1186/s13100-016-0066-8 (PMC4859993; doi:10.1186/s13100-016-0066-8)
Supplement: Additional file 6: — VL30 elements associated with Krueppel-associated box (KRAB) zinc finger proteins. The table provides information about all VL30 elements associated with KRAB zinc finger proteins and their relative distance to TSS. (PDF 10 kb) [file 13100_2016_66_MOESM6_ESM.pdf]

Gene Name	VL30 Name/Distance to TSS
**5730507C01Rik**	12qA1-2-2 (-641417), RLTR1C-chr12-1 (-429395), RLTR1C-chr12-2 (+182737), RLTR6_Mm-chr12-1 (+280393), RLTR1C-chr12-3 (+281906), RLTR6_Mm-chr12-2 (+307502), 12qA1-2 (+604420)
**AK078446**	2qH4 (-47223)
**Gm10324**	13qB3-2 (+271944)
**Gm13051**	4qE1 (-45787)
**Gm13235**	4qE1-2 (-32771)
**Gm14420**	2qH4 (-211075)
**Gm3604**	13qB3-7 (+88879)
**Gm4983**	RLTR6_Mm-chr12-3 (-259744), RLTR1C-chr12-7 (-51824), 12qA1-3 (+14194)
**Prdm9**	RLTR6_Mm-chr17-1 (-29625)
**RP23-282C23.3**	4qE1 (+52398)
**Zfp229**	RLTR6_Mm-chr17-5 (+41122)
**Zfp51**	17qA32tr (+25812)
**Zfp53**	17qA32tr (-12824)
**Zfp568**	RLTR6_Mm-chr7-2 (-4304)
**Zfp599**	9qA3tr (+31810)
**Zfp600**	4qE1-2 (+189055)
**Zfp746**	6qB23tr (-148229)
**Zfp791**	8qC3 (-16620)
**Zfp808**	13qB3tr (-42041), 13qB3-7 (+164405)
**Zfp809**	9qA3tr (+2382)
**Zfp820**	RLTR6_Mm-chr17-5 (+70913)
